# Single-shot single-beam coherent Raman scattering thermometry based on optically induced air lasing

**DOI:** 10.1038/s41377-024-01598-9

**Published:** 2024-11-25

**Authors:** Xu Lu, Yewei Chen, Francesco Mazza, Siyi He, Zihan Li, Shunlin Huang, Quanjun Wang, Ning Zhang, Bo Shen, Yuzhu Wu, Jinping Yao, Ya Cheng

**Affiliations:** 1 State Key Laboratory of High Field Laser Physics, Shanghai Institute of Optics and Fine Mechanics, Chinese Academy of Sciences, Shanghai, 201800 China; 2 Center of Materials Science and Optoelectronics Engineering, University of Chinese Academy of Sciences, Beijing, 100049 China; 3 School of Optical-Electrical and Computer Engineering, University of Shanghai for Science and Technology, Shanghai, 200093 China; 4 Faculty of Aerospace Engineering, Delft University of Technology, Kluyverweg 1, 2629 HS Delft, The Netherlands; 5 Institute of Modern Optics, Nankai University, Tianjin, 300350 China; 6 School of Microelectronics, Shanghai University, Shanghai, 200444 China

**Keywords:** Ultrafast photonics, Optical spectroscopy, Nonlinear optics

## Abstract

Thermometric techniques with high accuracy, fast response and ease of implementation are desirable for the study of dynamic combustion environments, transient reacting flows, and non-equilibrium plasmas. Herein, single-shot single-beam coherent Raman scattering (SS-CRS) thermometry is developed, for the first time to our knowledge, by using air lasing as a probe. We show that the air-lasing-assisted CRS signal has a high signal-to-noise ratio enabling single-shot measurements at a 1 kHz repetition rate. The SS-CRS thermometry consistently exhibits precision of <2.3% at different temperatures, but the inaccuracy grows with the increase in temperature. The high measurement repeatability, 1 kHz acquisition rate and easy-to-implement single-beam scheme are achieved thanks to the unique temporal, spectral and spatial characteristics of air lasing. This work opens a novel avenue for high-speed CRS thermometry, holding tremendous potential for fast diagnostics of transient reacting flows and plasmas.

## Abstract (web summary)

Single-shot single-beam coherent Raman scattering thermometry is developed by using air lasing as a probe. It shows the ability of single-shot measurements at 1 kHz and the precision of <2.3%.

## Graphical abstract

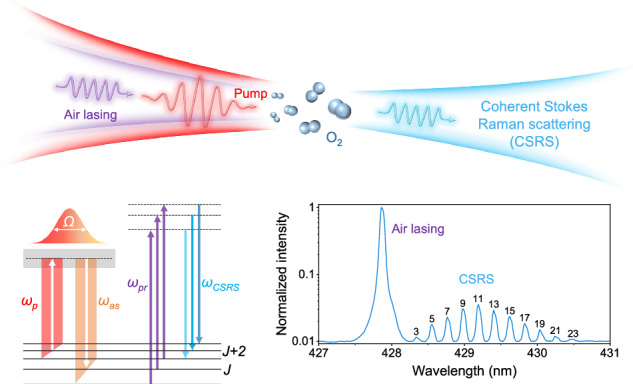

## Introduction

Determination of temperature and species concentration is of vital importance to unravel the underlying physical and chemical processes in reacting flows and plasmas, which are ubiquitous in the defense, energy, space sectors, as well as in various other research fields^1–3^. The precise measurement of these key parameters stands as an indispensable task for the efficient and clean utilization of fuels, the design and optimization of aero-engines, etc. However, combustion diagnostics is often implemented in complex, and even extreme scenarios, wherein rapid variations of temperature and composition of the gas medium pose substantial challenges to metrology. Coherent Raman scattering (CRS) spectroscopy has been proved to be a powerful non-intrusive tool in thermometry^4–9^ and species detection^7–11^, as it provides a strong, well-collimated signal as compared to spontaneous Raman scattering^12^. Particularly, hybrid femtosecond/picosecond (fs/ps) CRS, wherein the impulsively-excited Raman coherence is interrogated by a ps pulse, has been widely applied in combustion diagnostics^3^. The narrowband probe allows one to directly record vibrational or rotational fingerprints of Raman-active molecules in the spectral domain, providing the possibility of single-shot measurements at kilohertz, or even higher, rates^13^.

Nonetheless, most implementations of CRS spectroscopy necessitate two or more laser beams, where fine spatio-temporal control is essential to meet the phase-matching condition and suppress the non-resonant background^3^. In high-temperature, high-pressure turbulent environments, the intensity of CRS signals can be significantly reduced due to phase mismatch caused by beam jitter or refractive-index change. Single-beam CRS schemes, which have been widely studied^14–19^, can surmount these obstacles. Nevertheless, in conventional single-beam implementations, the probe is usually extracted from the fs pump pulse and their frequencies are very close, resulting in a poor signal-to-noise ratio (SNR). It is thus difficult to acquire either pure-rotational or vibrational CRS signals in single-shot acquisition mode. Besides, the ps probe is usually generated by pulse shaping using a spatial light modulator (SLM). Aside from the complexity of the apparatus, the low damage threshold of SLM ultimately limits the fs pulse energy and thus makes it difficult to realize single-shot measurements at 1 kHz or higher rate. Up to date, single-shot single-beam coherent Raman scattering (SS-CRS) thermometry remains a big challenge, deserving further exploration.

Air lasing has emerged as a remarkable phenomenon in the realm of ultrafast optics^20–22^ and, due to its inherent characteristics, provides an attractive alternative for the ps probe of CRS. In the time domain, air lasing typically exhibits a rapid rise and a slow decay, along with an intrinsic delay with respect to the pump pulse^23^. The asymmetric temporal envelope favors an efficient Raman scattering, while suppressing the non-resonant four-wave mixing background. Furthermore, the spectral bandwidth of air lasing can be as narrow as a few inverse centimeters^23^, guaranteeing a high spectral resolution even in pure-rotational CRS. Most importantly, the air lasing emission is coherently created along the plasma channel of the pump laser, thus automatically overlapping with the pump beam. Therefore, air lasing is an ideal light source of the single-beam CRS. Over the past years, various air-lasing-based CRS spectroscopies have been developed^23–30^ and applied to the high-sensitive detection of greenhouse gases^26,27^ and isotope identification^27,29^. Different from the aforesaid works for gas sensing, the main concerns of CRS thermometry for combustion diagnostics include high accuracy, fast instrument response and ease of implementation. Thus, aside from single-beam scheme, single-shot measurement is further required, which has been rarely explored.

In this work, single-shot single-beam CRS thermometry is developed based on optically induced air lasing. To the best of our knowledge, this is the first realization of 1 kHz single-shot CRS thermometry with a single fs laser beam. Through SS-CRS thermometry, the temperature in the interaction region is retrieved by fitting the measured rotational CRS spectrum to the theoretical spectrum. Alongside its ease of implementation, this air-lasing-based thermometry also shows high measurement repeatability and fast response time. Therefore, it provides an advanced tool for combustion diagnostics in harsh scenarios and non-equilibrium systems^31^.

## Results

### Basic principle of SS-CRS thermometry based on air lasing

**Fig. 1:**
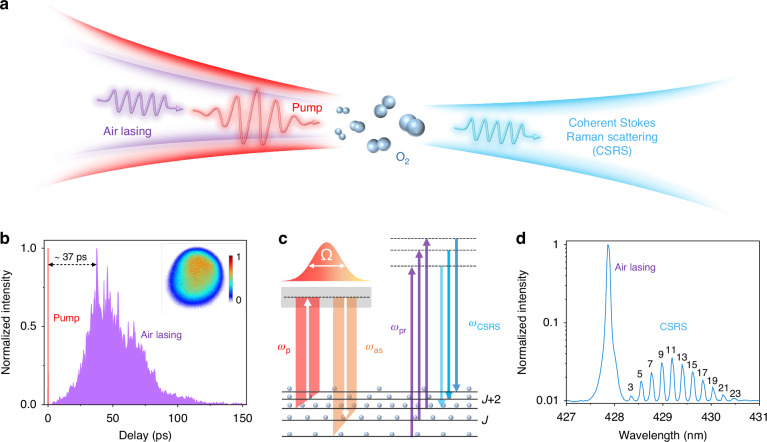
Basic principle of SS-CRS thermometry based on optically induced air lasing. **a** Schematic of basic processes of SS-CRS thermometry based on air lasing. **b** Temporal envelope of ${{\rm{N}}}_{2}^{+}$ lasing and the delay of lasing emission with respect to the pump laser. The inset shows the spatial profile of the ${{\rm{N}}}_{2}^{+}$ lasing. **c** Energy-level diagram of rotational coherent Stokes Raman scattering (RCSRS). ${\omega }_{p}$, ${\omega }_{{as}}$, ${\omega }_{{pr}}$, and ${\omega }_{{CSRS}}$ represent, respectively, the angular frequencies of the pump, the anti-Stokes, the probe and the CSRS fields. $\varOmega$ represents the frequency difference between the pump and anti-Stokes fields. The incident pump laser provides both the pump and anti-Stokes fields, whereas the ${{\rm{N}}}_{2}^{+}$ lasing serves as the probe field. **d** The typical RCSRS spectrum measured in ${{\rm{O}}}_{2}$ at room temperature. In order to measure the air lasing and RCSRS signal simultaneously, a filter was used to attenuate the air lasing signal in the data acquisition. Each spectral line is labeled by the rotational quantum number of lower state ($J$) in the corresponding Raman transition ($J+2\to J$)

SS-CRS is essentially a resonant four-wave-mixing process, wherein the molecular Raman coherence is impulsively excited by a broadband fs laser and subsequently probed by the narrowband ps air lasing, as shown in Fig. 1a. The fs laser, after ionizing molecular nitrogen (${{\rm{N}}}_{2}$), serves as a degenerate pump and anti-Stokes pulse, which can coherently excite the rotation of the target oxygen molecule (${{\rm{O}}}_{2}$). The air lasing emission at ~428 nm, corresponding to the transition between the ${B}^{2}{\Sigma }_{{\rm{u}}}^{+}(\nu ^{\prime} =0)$ and ${X}^{2}{\Sigma }_{{\rm{g}}}^{+}(\nu =1)$ states of molecular nitrogen ion (${{\rm{N}}}_{2}^{+}$), acts as a ps probe. The temporal envelope of ${{\rm{N}}}_{2}^{+}$ lasing, obtained by cross-correlation measurements, is illustrated in Fig. 1b. When the ${{\rm{N}}}_{2}^{+}$ lasing interacts with the coherently rotating molecules, the rotational coherent Stokes Raman scattering (RCSRS) signal is efficiently generated. The energy-level diagram for this process, and a typical RCSRS spectrum of ${{\rm{O}}}_{2}$ are shown in Fig. 1c, d, respectively.

The different rotational Raman transitions are clearly resolved in the measured spectrum thanks to the narrow spectral bandwidth (<4 cm^−1^) of the air lasing emission. The strength of each rotational Raman peak is closely related to the population difference between the initial and final rotational levels. The rotational population itself depends on the gas temperature via Boltzmann distribution. Hence, we can retrieve the temperature in the interaction region from the RCSRS spectrum by fitting the measured result with the theoretical spectra calculated at different temperatures. Moreover, the measured spectrum shows a good SNR, which is attributed to the efficient rotational excitation provided by the fs laser and the unique spatio-temporal envelope of the ${{\rm{N}}}_{2}^{+}$ lasing. As indicated in Fig. 1b, the ${{\rm{N}}}_{2}^{+}$ lasing has an inherent delay of ~37 ps with respect to the pump laser. The probe pulse delay ($\tau$) here is defined as the time interval from the center of the pump pulse profile to the peak of ${{\rm{N}}}_{2}^{+}$ lasing profile. The temporal separation of two pulses avoids generation of non-resonant four-wave mixing, which may interfere with the RCSRS signal or even overwhelm it. The ${{\rm{N}}}_{2}^{+}$ lasing furthermore shows a near-Gaussian spatial profile, as illustrated in the inset of Fig. 1b. The spatial overlap between the pump and probe pulses is essential to efficient generation of the CRS signal. Since the pump pulse has a Gaussian profile, the near-Gaussian profile of the ${{\rm{N}}}_{2}^{+}$ lasing optimizes their overlap and guarantees a good SNR of the CRS signal. The combination of the fs pump pulse and the self-generated ps air lasing enables the single-shot, single-beam operation of CRS thermometry.

### Experimental demonstration and performance of SS-CRS thermometry

**Fig. 2:**
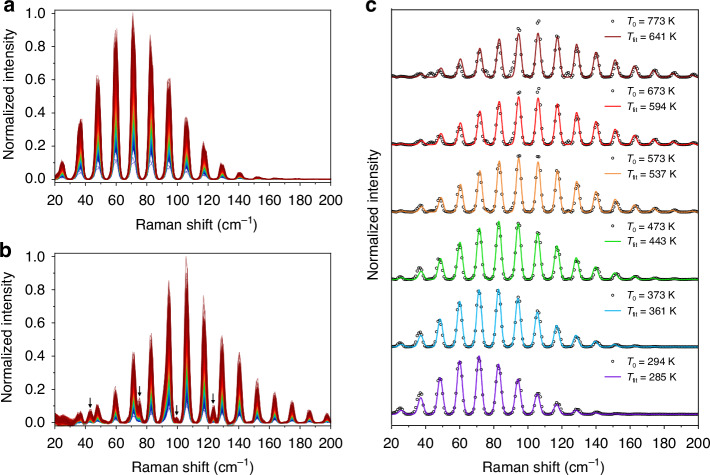
Typical RCSRS spectra measured at different temperatures and the best-fit results. **a** The 1000 consecutive single-shot RCSRS spectra measured at the temperature of 294 K. **b** The 1000 consecutive single-shot RCSRS spectra measured at the temperature of 773 K. The baseline of each single-shot spectrum was subtracted and the 1000 spectra were normalized by the strongest one at the same temperature. The black arrows indicate RCSRS spectral lines from ${{\rm{N}}}_{2}$. **c** The averages of the 1000 single-shot spectra acquired at different target temperatures (black circles) and corresponding best-fit theoretical spectra (solid lines). The target temperature ${T}_{0}$ in the tube furnace and the best-fit temperature ${T}_{{\rm{fit}}}$ are labeled on the right side of each spectrum

Single-shot RCSRS spectra were experimentally acquired in the furnace at a 1 kHz rate. By way of example, the 1000 consecutive single-shot Raman spectra measured at the furnace temperature of 294 K and 773 K are shown in Fig. 2a, b, respectively. For illustration purpose only, the averages of the 1000 single-shot spectra acquired at each temperature setpoint are shown in Fig. 2c (black circles), and compared to the best-fit theoretical spectra (solid lines). These spectra captured at different temperatures were normalized independently, and the corresponding baseline was subtracted. It is worth stressing here that these averaged spectra are not equal to the spectrum captured over the 1 s exposure time. The underling 1000 single-shot spectra were independently fitted to measure the temperature at each furnace setpoint, as discussed later.

As clearly seen in Fig. 2, each RCSRS spectrum consists of a series of equally spaced peaks. Their frequency shift with respect to the ${{\rm{N}}}_{2}^{+}$ lasing, i.e., Raman shift, is in good agreement with the approximate theoretical formula $\Delta \widetilde{{\rm{\lambda }}}=(4J+6){\rm{B}}$, where ${\rm{B}}=1.43$ ${{\rm{cm}}}^{-1}$ is the rotational constant of ${{\rm{O}}}_{2}$ molecule^32^, and $J$ is the rotational quantum number of the lower rotational state. As the temperature increases, the measured RCSRS spectrum covers a broader range and the strongest Raman line shifts towards transition between higher rotational states. These variations mirror the underlying change in the rotational population as a function of temperature, as per the Boltzmann statistics. As the temperature increases, the maximum population shifts towards the higher rotational state. Moreover, since the gas pressure of furnace chamber was kept at 50 mbar at all temperature setpoints, a weaker RCSRS signal from ${{\rm{O}}}_{2}$ was recorded at higher temperature due to the decrease in gas density. Nonetheless, it can be seen that, even at the highest target temperature (773 K), the SNR of the measured RCSRS spectra is high enough to perform single-shot measurement. Nonetheless, a signal contamination caused by ${{\rm{N}}}_{2}$ in the first gas chamber is observed in Fig. 2b. As this chamber was maintained at room temperature, the relative contribution of the ${{\rm{N}}}_{2}$ RCSRS signal became significant at the highest temperature.

As mentioned, the envelopes of the experimental spectra in Fig. 2c qualitatively reflect the temperature variation in the interaction region. Quantitative results were obtained by fitting the measured spectra to the theoretical spectra calculated by the theoretical model given in Materials and methods, thus estimating the gas temperature in the interaction region. As shown in Fig. 2c, the fitted spectra reproduce the main features of the measured RCSRS spectra, including the relative intensity of all Raman peaks as well as the spectral position and width of each peak. The residuals between theoretical and experimental spectra are very small except for 1 ~ 2 Raman peaks at temperatures above 500 K.

**Fig. 3:**
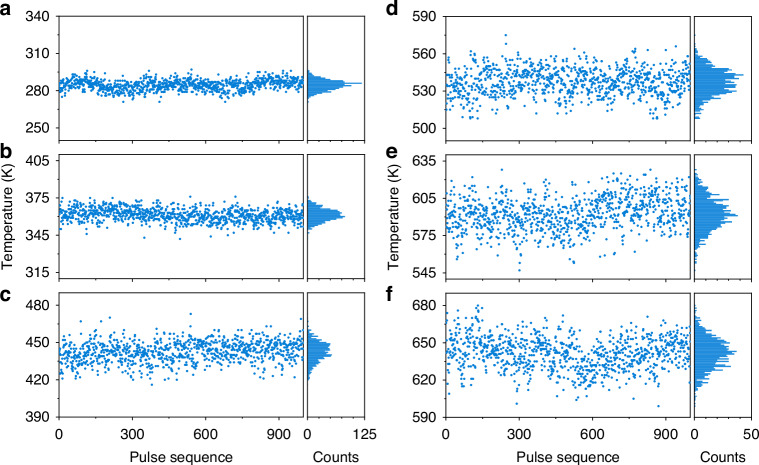
The best-fit temperatures obtained by the measured 1000 consecutive single-shot RCSRS spectra and the corresponding histograms at different furnace temperatures. **a**–**f** The results of furnace temperature 294 K, 373 K, 473 K, 573 K, 673 K and 773 K, respectively

**Fig. 4:**
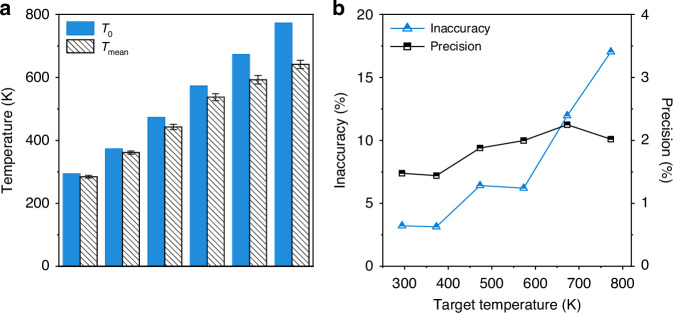
The inaccuracy and precision of SS-CRS thermometry. **a** Comparison between the best-fit temperature extracted from the single-shot RCSRS spectra and the target temperature. The error bars represent the standard deviation ${T}_{{\rm{std}}}$. **b** The inaccuracy and precision of SS-CRS thermometry as a function of the target temperature

The temperature dynamics measured by 1000 consecutive single-shot CSRS spectra, recorded over a 1 s acquisition window at each furnace temperature setpoint are shown in Fig. 3. These results clearly illustrate the ability of SS-CRS to monitor temperature changes at a high repetition rate. The corresponding histograms of the best-fit temperatures are furthermore given in the right panels. The temperature step was taken as 1 K in the fitting process. It can be clearly seen that these histograms approximately obey the normal distribution with different mean values and standard deviations. At room temperature (i.e., 294 K), the best-fit temperatures are concentrated in the vicinity of 286 K. As the target temperature grows, the best-fit temperatures of 1000 single-shot spectra show a broader distribution. To perform quantitative analysis, Fig. 4a compares the average value ${T}_{{\rm{mean}}}$ of the best-fit temperatures extracted from 1000 single-shot spectra to the target temperature ${T}_{0}$ in the tube furnace. The standard deviation ${T}_{{\rm{std}}}$ of the best-fit temperatures is marked by an error bar. Figure 4b shows the inaccuracy and precision of SS-CRS thermometry as a function of the target temperature. According to the commonly-used characterization parameters in CRS thermometry^8^, we quantify the measurement inaccuracy of our SS-CRS technique by the relative error $\left|{T}_{0}-{T}_{{\rm{mean}}}\right|/{T}_{0}$, while its precision is represented by the relative standard deviation ${T}_{{\rm{std}}}/{T}_{{\rm{mean}}}$.

The quantitative results in Fig. 4a show that the best-fit temperature gradually deviates from the target temperature as the temperature grows. As quantified in Fig. 4b, the measurement inaccuracy increases to 17.0% at 773 K from 3.2% at room temperature. The significant increase of measurement error at high temperature could be primarily attributed to the simplified pump pulse envelope, the neglected propagation effect in the theoretical model, and the contamination by the N_2_ RCSRS signal. A detailed discussion of these issues is given in the following section. Contrasted to its inaccuracy, the precision of our SS-CRS thermometry is <2.3% at all temperatures, as indicated by the black squares in Fig. 4b. This value is comparable with, if not better than, that of most laser diagnostics techniques^33–36^, some of which cannot achieve single-shot thermometry at 1 kHz, or lack in the spatial resolution.

## Discussion

The above results demonstrate the feasibility of 1 kHz, single-shot single-beam CRS measurements based on optically induced air lasing. This state-of-the-art thermometry ingeniously combines the advantages of the fs pump laser and the self-generated air lasing. Especially, the time-frequency characteristics of the air lasing emission endow this technique with the capability of single-shot, single-beam measurement. This novel scheme not only greatly simplifies the implementation of CRS thermometry, but also allows for combustion diagnostics in harsh environments or at standoff locations. It benefits from the inherent spatial overlap of the pump, anti-Stokes and probe pulses and from the elimination of non-resonant background due to the intrinsic delay of the pump pulse and air lasing emission.

As the first proof-of-principle demonstration, both accuracy and precision of SS-CRS thermometry reach a satisfactory level at temperatures up to 100 °C^8^. Nevertheless, the accuracy degrades at higher temperatures, as shown in Fig. 4b. Several factors could be responsible for this.

Firstly, in the present simulation the pump pulse was assumed to be a Gaussian pulse. Nevertheless, the nonlinear propagation of the high-energy pump laser in the gas medium can cause the variation of its temporal profile due to self-phase modulation and gas ionization effects. This would influence the Raman excitation^9^ and lead to deviations from the theoretical spectra and a bias in the calculated temperature. Moreover, the gas pressure in the furnace was kept at 50 mbar at all temperature setpoints. The subsequent decrease in gas density with the increasing temperature deteriorates the SNR of the RCSRS signal and probably affects the spatio-temporal characteristics of the pump pulse, which could also cause the measurement error. The impact of the pulse propagation in reactive environments on Raman excitation has been recently investigated, and an experimental procedure for its in-situ referencing was proposed^37^, which could mitigate the influence of the pulse propagation in an equivalent two-beam CRS scheme. Alternatively, the temporal profile of the pump pulse after propagation can be experimentally measured via frequency-resolved optical gating. We expect a better characterization of the Raman excitation efficiency in the experiment to reduce the fitting error and improve the measurement accuracy.

Secondly, it should be noted that the target temperature ${T}_{0}$ was measured by a thermocouple with standard calibration. In non-standard environments, however, the absolute values of thermocouple measurement can be affected by various factors like heat transfer. The measurements were conducted under incomplete adiabatic conditions and the gas in the furnace tube had a temperature gradient. Therefore, there is a certain deviation of the nominal ${T}_{0}$ from the true temperature in the furnace, which is especially true when the gas is heated to high temperature.

Last but not least, ${{\rm{N}}}_{2}$ molecules are inevitably excited during the ${{\rm{N}}}_{2}^{+}$ lasing generation. Contamination of the ${{\rm{O}}}_{2}$ RCSRS spectra by a weak ${{\rm{N}}}_{2}$ RCSRS signal could also contribute to the reported inaccuracy. This issue can be readily solved in the future SS-CRS implementation by introducing a spectral filter before the measurement region. It is also noteworthy that what is here reported as a signal contamination is at the same time showcasing a great feature of the CRS technique, namely, the ability to simultaneously interrogate all the Raman-active species present in the detection region. The complex chemistry of a reacting flow can thus be studied by a single diagnostic technique. In this sense, SS-CRS thermometry could be complemented in the future by simultaneous concentration measurements, all realized by using a single laser source. This can be easily achieved by introducing spectroscopic models for different Raman-active molecules in the calculation of theoretical spectra.

Future work should improve the accuracy of the temperature measurement, by considering or minimizing the influences of aforementioned factors, for practical applications in high-temperature, high-pressure combustion scenarios. Indeed, the SS-CRS thermometry demonstrated here will show major advantages when applied to the study of dynamic combustion environments or high-speed reacting flows, where steep turbulence and refractive index gradients dramatically change on short timescales. The single-beam scheme avoids the need for complex operations and synchronization of multiple laser beams, having great potential for improving the stability of high-speed measurement in harsh scenarios. The single-beam RCSRS thermometry demonstrated here is suitable for high-precision temperature measurement between 300 K and 1200 K. However, the technique can be extended to vibrational CRS thermometry to realize thermometry at higher temperatures with sufficient precision. The possible overlap of the rotational Raman spectra of different molecules can also be well solved by using the vibrational CRS thermometry, where the spectral signatures of different chemical species are more easily discriminated.

When it comes to the diagnostics of turbulent combustion fields with temperature gradients, the spatial resolution of a thermometric technique plays a crucial role, and the spatial averaging effects could lead to significant measurement biases^38^. In the present single-beam scheme, the collinear configuration of the air lasing emission and the fs pump laser limits the longitudinal spatial resolution. The temperature extracted from the RCSRS spectra is the integral result over the interaction region. In other words, the spatial resolution is determined by the focusing geometry. If a tighter focusing lens or a telescope system is used, the spatial resolution can be further improved. A spatial resolution even comparable to that of a BOXCARS configuration^5^ could be achieved by using the spatio-temporal focusing scheme^39^.

An additional point worth noting in the current scheme is the energy of the ${{\rm{N}}}_{2}^{+}$ lasing emission. As compared to ps sources commonly employed in the fs/ps CRS scheme^5,8^, the energy of the ${{\rm{N}}}_{2}^{+}$ lasing emission is relatively low, usually on the order of a few nanojoules per pulse. While the SNR of the RCSRS signal reported in the present work is sufficient to achieve single-shot measurements, the use of sheet optics to realize single-shot 1D spectroscopic imaging^40^ requires a higher SNR. Alternatively, temperature distributions could be measured by scanning the region of interest or employing spatially-multiplexed probing schemes^41^. These technical improvements will help us greatly improve the spatial resolution, the response speed, the measurement accuracy and precision of SS-CRS thermometry, and will be considered in our future work. We believe that the air-lasing-based thermometry with further refinement can be applied in the study of dynamic high-temperature combustion environments, transient reacting flows, and non-equilibrium plasmas.

In summary, we developed 1 kHz single-shot coherent Raman thermometry with a single femtosecond laser beam. This single-shot, single-beam thermometric technique fully utilizes the spatial, spectral and temporal properties of air lasing. The single-beam scheme largely simplifies the apparatus, and overcomes the difficulty of the spatio-temporal control of multiple beams in high-temperature turbulent environments. It is proven that the inaccuracy and precision of the thermometry reach 3.2% and 1.5% at room temperature, respectively, while operating in the 1 kHz, single-shot acquisition mode. As the temperature increases, the precision remains almost unchanged, whereas the accuracy worsens. Although the accuracy is not satisfactory at high temperatures in the present stage, some technical improvements promise to overcome this limitation. Air-lasing-based SS-CRS thermometry will show promising applications in the investigation of reacting flows and plasmas.

## Materials and methods

### Experimental details

The experiment was carried out employing a commercial Ti:sapphire laser system (Legend Elite-Duo, Coherent), which delivers ~40 fs laser pulses centered at 800 nm at 1 kHz repetition rate. The laser pulse with an energy of ~6 mJ was focused by an *f* = 60 cm lens into a gas chamber filled with ${{\rm{N}}}_{2}$ gas at 5 mbar. In this way, ${{\rm{N}}}_{2}^{+}$ lasing was coherently created along the plasma channel, giving rise to the narrowband coherent emission at ~428 nm. The ${{\rm{N}}}_{2}^{+}$ lasing, together with the residual fs laser, was then focused by an *f* = 40 cm concave mirror into 50 mbar ${{\rm{O}}}_{2}$ gas to produce the RCSRS signal. In order to demonstrate high-temperature SS-CRS, a tube furnace was designed to heat the ${{\rm{O}}}_{2}$ molecules, and thermocouples were applied for the measurement and feedback control of the temperature. The temperature of the heated gas could be controlled automatically around the set target temperature. The thermostatic zone was longer than 15 cm in the tube furnace, and the RCSRS signal was generated in this region. After the furnace chamber, the residual fs laser and supercontinuum emission were suppressed by a dichroic mirror, two pieces of blue glasses and a short-pass filter. The RCSRS signal of ${{\rm{O}}}_{2}$ molecules was further filtered by a long-pass filter and detected by a spectrometer (Shamrock 500i, Andor) equipped with a 2400 grooves/mm grating and an intensified CCD detector (iStar, Andor). Crop mode acquisition was adopted to realize single-shot measurement at the 1 kHz rate.

### Theoretical model

As described above, CRS is a resonant four-wave mixing process. The corresponding third-order polarization can be simplified as^5,42^

1 $${P}^{(3)}\left(t,\tau \right)={\left(\frac{i}{{{\hslash }}}\right)}^{3}{E}_{{pr}}\left(t\right){\int }_{0}^{{{\infty }}}d{t}^{{\prime} }R({t}^{{\prime} }){E}_{p}^{* }\left(t+\tau -{t}^{{\prime} }\right){E}_{p}(t+\tau -{t}^{{\prime} })$$

Here, ${E}_{p}$ represents the electric field envelope of the pump pulse, which was assumed to be a Gaussian pulse with a full-width-at-half-maximum of 44 fs, considering the dispersion of the window and lens. ${E}_{{pr}}$ is the electric field envelope of the probe pulse. The temporal profile of the probe pulse, and its delay $\tau$ with respect to the pump pulse, were determined by the cross-correlation measurement result in Fig. 1b. The non-resonant term was neglected in Eq. (1) owing to temporal separation of the pump and probe pulses.

The molecular response function is expressed as $R\left(t\right)=\sum \nolimits_{n,m}I_{nm}\left(T\right){e}^{{-i\omega_{nm}} t-\Gamma_{nm} t}$ with transition frequency $\omega_{nm}$, collision-dominated Raman linewidth $\Gamma_{nm}$, and transition strength $I_{nm}\left(T\right)$^5,42^. The summation is performed for all of the possible energy states occupied by the Raman-active molecules. The Raman transition frequencies are defined as the energy difference between the initial and final rotational states, and were calculated using the molecular parameters accessible in the reference^32^. For ${{\rm{O}}}_{2}$, each energy state is a triplet, due to the coupling of electron spin and nuclear angular momentum^43^. In the present work, the model is simplified by considering the triplets as degenerate states. Collisional effects are also ignored, i.e., $\Gamma_{nm} =0$, due to the relative low gas pressure in the furnace chamber. The transition strength is related to the Raman transition cross section and the population difference $\Delta \rho$ between the upper and lower rotational states. $\Delta \rho$ is a function of temperature $T$ based on Boltzmann distribution. As a result, the temperature can be extracted by comparing the experimentally-measured RCSRS spectrum with the theoretically-calculated one. Theoretical spectra at different temperatures are obtained by convolving ${\left|{\int }_{-{{\infty }}}^{+{{\infty }}}{P}^{(3)}\left(t,\tau \right)\exp (i\omega t){dt}\right|}^{2}$ with the instrument response function.

## Data Availability

Data underlying the results presented in this paper are not publicly available at this time but may be obtained from the authors upon reasonable request.
